# Modulation of habenular and nucleus accumbens functional connectivity by ketamine in major depression

**DOI:** 10.1002/brb3.3511

**Published:** 2024-06-19

**Authors:** Brandon Taraku, Joana R. Loureiro, Ashish K. Sahib, Artemis Zavaliangos‐Petropulu, Noor Al‐Sharif, Amber M. Leaver, Benjamin Wade, Shantanu Joshi, Roger P. Woods, Randall Espinoza, Katherine L. Narr

**Affiliations:** ^1^ Ahmanson‐Lovelace Brain Mapping Center, Department of Neurology University of California Los Angeles Los Angeles California USA; ^2^ Department of Radiology Northwestern University Chicago Illinois USA; ^3^ Division of Neuropsychiatry and Neuromodulation Massachusetts General Hospital and Harvard Medical School Boston Massachusetts USA; ^4^ Department of Psychiatry and Biobehavioral Sciences University of California Los Angeles Los Angeles California USA

**Keywords:** fMRI, habenula, ketamine, nucleus accumbens, reward

## Abstract

**Introduction:**

Major depressive disorder (MDD) is associated with dysfunctional reward processing, which involves functional circuitry of the habenula (Hb) and nucleus accumbens (NAc). Since ketamine elicits rapid antidepressant and antianhedonic effects in MDD, this study sought to investigate how serial ketamine infusion (SKI) treatment modulates static and dynamic functional connectivity (FC) in Hb and NAc functional networks.

**Methods:**

MDD participants (*n* = 58, mean age = 40.7 years, female = 28) received four ketamine infusions (0.5 mg/kg) 2−3 times weekly. Resting‐state functional magnetic resonance imaging (fMRI) scans and clinical assessments were collected at baseline and 24 h post‐SKI. Static FC (sFC) and dynamic FC variability (dFCv) were calculated from left and right Hb and NAc seeds to all other brain regions. Changes in FC pre‐to‐post SKI, and correlations with changes with mood and anhedonia were examined. Comparisons of FC between patients and healthy controls (HC) at baseline (*n* = 55, mean age = 32.6, female = 31), and between HC assessed twice (*n* = 16) were conducted as follow‐up analyses.

**Results:**

Following SKI, significant increases in left Hb‐bilateral visual cortex FC, decreases in left Hb‐left inferior parietal cortex FC, and decreases in left NAc‐right cerebellum FC occurred. Decreased dFCv between left Hb and right precuneus and visual cortex, and decreased dFCv between right NAc and right visual cortex both significantly correlated with improvements in mood ratings. Decreased FC between left Hb and bilateral visual/parietal cortices as well as increased FC between left NAc and right visual/parietal cortices both significantly correlated with improvements in anhedonia. No differences were observed between HC at baseline or over time.

**Conclusion:**

Subanesthetic ketamine modulates functional pathways linking the Hb and NAc with visual, parietal, and cerebellar regions in MDD. Overlapping effects between Hb and NAc functional systems were associated with ketamine's therapeutic response.

## Graphical abstract

We investigated how ketamine treatment perturbs static and dynamic functional connectivity from reward circuitry in treatment resistant depression. We found that ketamine produces functional connectivity changes between the habenula and visual cortex, and changes in dynamic functional connectivity between the habenula, nucleus accumbens, visual cortex, and posterior cingulate cortex were associated with greater treatment response.

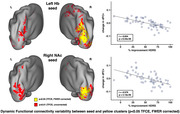

## 1 INTRODUCTION

Major depression, characterized by low mood and a loss of pleasure and interest in activities or anhedonia among other symptoms, affects at least 5% of adults worldwide (Institute for Health Metrics and Evaluation, 2019). Though treatable, only one‐third of patients remit after receiving first‐line antidepressants that take weeks to months to induce clinical benefits. About 30−40% of patients still fail to remit (Gaynes et al., 2009; Warden et al., 2007) after multiple treatment trials and are characterized as having treatment‐resistant depression (TRD). Anhedonia, a core symptom of depression, has been linked with deficits in reward processing regions of the brain (Gorwood, 2008; Nestler, 2015). Even among patients who remit or respond to standard antidepressants, anhedonic symptoms often persist (Cao et al., 2019; Treadway & Zald, 2011). Anhedonia and reward processing deficits are shown to predict treatment resistance (Morris et al., 2009; Uher et al., 2012), risk of suicide (Ducasse et al., 2018; Fawcett et al., 1990; Winer et al., 2014), and negatively impact quality of life in people with depression (Barthel et al., 2020; Ritsner et al., 2011).

At a subanesthetic dose, ketamine, a N‐methyl‐D‐aspartate (glutamate) receptor (NMDAR) antagonist, is shown to rapidly reduce depressive and suicidal symptoms in patients with TRD. Though antidepressant response is typically transient (∼1 week) (Duman, 2018; Kraus et al., 2019; McIntyre et al., 2021; Zarate et al., 2006), improvements in anhedonia appear to outlast and be independent of overall antidepressant outcomes (Lally et al., 2014; Nogo et al., 2022; Pulcu et al., 2022; Wilkowska et al., 2021). Moreover, ketamine treatment is shown to result in improvements in anticipatory, consummatory, and motivation‐related reward processes that are considered dimensions of anhedonia in humans and in animal models (Nogo et al., 2022). Though the molecular mechanisms of ketamine's antidepressant action are not yet resolved, available data support that NMDAR‐related signaling pathways induce rapid dendritic and synaptic remodeling (Duman, 2018; Duman et al., 2019; Kraus et al., 2019). These processes of neural plasticity are assumed to then evolve to higher brain systems‐level reorganization to influence behavior, which include modulation of downstream monoaminergic neurotransmitter systems targeted by standard antidepressants (Can et al., 2016; Gigliucci et al., 2013; Kokkinou et al., 2018; Li et al., 2015; Witkin et al., 2016). Preclinical studies further suggest that ketamine's therapeutic effects are at least partially mediated by the mesolimbic system (Cardona‐Acosta & Bolaños‐Guzmán, 2023). The mesolimbic pathway, connecting the midbrain and ventral tegmental area (VTA) with the ventral striatum/nucleus accumbens (NAc) and other limbic and cortical areas, is repeatedly shown to drive reward‐related behavior (Berridge & Kringelbach, 2015; Gorwood, 2008; Nestler, 2015; Pool et al., 2022). The NAc, a key node of dopaminergic reward circuitry, is widely implicated in the pathophysiology of depression (Shirayama & Chaki, 2006; Xu et al., 2020) and mediates positive valence‐related emotional response and reward processing (Epstein et al., 2006; Gorwood, 2008; Heller et al., 2009; Keedwell et al., 2005; Liu et al., 2021; Pizzagalli et al., 2009; Pool et al., 2022; Salgado & Kaplitt, 2015). Furthermore, functional connectivity (FC) between the NAc and prefrontal cortex is shown to relate to anhedonia in depression (Heller et al., 2009; Liu et al., 2021) and antidepressant response (Heller et al., 2013).

A key region involved in reward that is also relevant to the therapeutic mechanisms of ketamine is the habenula (Hb) located near the posterior and medial region of the thalamus (Hikosaka et al., 2008). The lateral component of the habenula (LHb) projects to dopaminergic VTA and serotonergic raphe nucleus, where it inhibits monoaminergic activity, therefore‐ regulating reward behavior (Lecca et al., 2014; Thompson, 2023) and is often referred to as the “antireward” system (Lecca et al., 2014; Matsumoto & Hikosaka, 2007). Preclinical and human studies strongly implicate LHb dysfunction as contributing to depression and anhedonia, and suggest its involvement in antidepressant response (Barreiros et al., 2022; Browne et al., 2018; Gold & Kadriu, 2019; Lecca et al., 2014; Thompson, 2023). Furthermore, the blockade of LHb NMDAR bursting activity is specifically linked with the rapid antidepressant action of ketamine in preclinical models (Yang et al., 2018; Yang et al., 2018). In humans, a recent resting‐state fMRI (rsfMRI) study reported that increased FC between the Hb and frontal pole, occipital pole/cortex, temporal pole, and parahippocampal gyrus associated with antidepressant response following single‐dose ketamine (Rivas‐Grajales et al., 2021). Another study found that ketamine response in major depressive disorder (MDD) is associated with changes in FC between the Hb and midbrain (VTA) and brainstem nuclei (SN) (Chen et al., 2024).

To further understand how ketamine modulates antidepressant and reward‐related brain circuitry, the current investigation addressed how FC between both the NAc and Hb to all regions across the brain are modulated by ketamine at the functional systems level. Disruptions in dynamic FC, which reflect temporal fluctuations in FC between brain regions over short intervals (Leonardi & Van De Ville, 2015; Liu & Duyn, 2013; Preti et al., 2017), have been implicated in MDD (Chen et al., 2022; Kaiser et al., 2016; Zhou et al., 2021; Zhou et al., 2022), and together with static FC (sFC) may offer and more complete view of resting‐state FC, such as how networks coalesce and dissolve over time (Kaiser et al., 2016). Therefore, changes in sFC as well as dynamic FC variability (dFCv), defined as the standard deviation of FC across a series of windowed time series throughout fMRI acquisition, were investigated here. To accomplish this goal, advanced rsfMRI acquisition and computational analysis methods were combined to determine whether Hb or NAc to whole brain FC change over time and associate with improvements in mood and anhedonia in TRD participants receiving serial ketamine infusion (SKI) treatment. Based on the limited prior literature (Abdallah et al., 2017; Chen et al., 2024; Rivas‐Grajales et al., 2021), we hypothesized that changes in NAc and Hb FC with frontal, temporal, and occipital regions and potentially measurable brainstem regions would change over time and associate with improved mood or anhedonia. We also compared Hb and NAc FC between patients scanned at baseline and nondepressed controls to assist in the interpretation of results, as well as examined the effects of time in a subsample of controls without treatment who were assessed twice at an interval similar to patients.

## 2 MATERIALS AND METHODS

### 2.1 Participants

Fifty‐eight depressed participants (average age = 40.7 years, 28 female) participated in this naturalistic clinical trial (NCT02165449) (https://clinicaltrials.gov/study/NCT02165449). Participants received four serial intravenous ketamine infusions over the course of 2 weeks. MRI scanning and clinical and behavioral data were acquired (1) prior to treatment (baseline), occurring < 1 week before the first ketamine infusion, and (2) 24 h after the last ketamine infusion.

Participants were included if they met criteria for TRD, which was defined as nonresponse to ≥2 antidepressant trials of adequate dosage and duration, and being continuously depressed for ≥6 months. Additional eligibility criteria for participants included DSM‐V‐defined major depression (First et al., 2015), moderate to severe depressive symptoms as per the Hamilton Depression Rating Scale (HDRS) 17‐item (scores ≥ 17) (Hamilton, 1960), no prior psychotic reactions to medications, alcoholic or illicit substances in the past, or other physical or clinical contraindications to ketamine. Exclusion criteria included any unstable medical or neurological condition, current substance abuse or dependence (measured by laboratory testing) or substance abuse history within the preceding 3 months, current or past history of psychosis, schizophrenia, intellectual disability or other developmental disorder, diagnosis of dementia, and any contraindication to scanning (e.g., metal implants or claustrophobia).

In addition, 55 healthy controls (HC) who were not depressed and that otherwise met the same exclusion criteria as patients were assessed at baseline (average age = 32.6, female = 31) and a subsample of 16 participants were assessed after approximately 2 weeks (average age = 28.8 years, 9 female). All subjects were recruited from the Los Angeles area through advertisements, clinician referral or clinicaltrials.gov. All subjects provided written informed consent following procedures approved by the UCLA Institutional Review Board.

### 2.2 Ketamine treatment

Ketamine treatment was administered 2−3 times a week and included intravenous pump delivery of a subanesthetic dose (0.5 mg/kg) of racemic ketamine diluted in 60 cc normal saline. Participants were permitted to remain on approved monoaminergic antidepressant therapy if the dosage was unchanged in the preceding 6 weeks. Benzodiazepines were discontinued > 72 h prior to all study visits including scanning sessions. Concurrent medications used by participants are listed in Table 1.

**TABLE 1:** Stable medications used by participants in ketamine treatment trial.

Current use of medications	*N*
SSRIs	18
SNRIs	18
MAOIs	2
Lithium	1
Benzodiazepines	15
Anticonvulsants	15
Typical antipsychotics	1
Atypical antipsychotics	12
Stimulants	13
Sleep medications	7
Opiates	1
None	12

*Note*: Antidepressant medications were required to be unchanged at least 6 weeks prior to enrollment and for the duration of the trial. Benzodiazepines were discontinued on the day of treatments and assessments.

Abbreviations: MAOIs, monoamine oxidase inhibitors; SNRIs, selective norepinephrine reuptake inhibitors; SSRIs, selective serotonin reuptake inhibitors.

### 2.3 Clinical assessments

At each time point, depression severity was assessed using the HDRS (Hamilton, 1960), and anhedonia was measured with the Snaith−Hamilton Pleasure Scale (SHAPS) (Snaith et al., 1995). Remitters were defined as participants with an HDRS score of ≤7 post‐SKI treatment.

### 2.4 Image acquisition

Imaging was performed on a Siemens 3T Prisma MRI system at the UCLA Brain Mapping Center using a 32‐channel head coil. Imaging sequences were identical to those used by the Human Connectome Project (HCP) Lifespan studies for Aging and Development (Harms et al., 2018). Structural sequences included T1‐weighted (voxel size [VS] = 0.8 mm isotropic; repetition time [TR] = 2500 ms; echo time [TE] = 1.81:1.79:7.18 ms; inversion time [TI] = 1000 ms; flip angle [34] = 8.0^°^; acquisition time [TA] = 8:22 min), and T2‐weighted acquisitions (VS = 0.8 mm isotropic; TR = 3200 ms; TE = 564 ms; TA = 6:35 min), both with real‐time motion correction (Tisdall et al., 2016). rsfMRI used a multiband EPI sequence with opposite phase encoding directions over two runs (VS = 2 mm isotropic; TR = 800 ms; TE = 37 ms, FA = 52^°^, MB accl. factor = 8; phase enc. direction = AP(run1)/PA (run2); TA = 13:22 min), along with two sets of spin echo images used for distortion correction (Andersson et al., 2003; Smith et al., 2004). During rsfMRI, subjects viewed a fixation cross.

### 2.5 Image processing and denoising

Imaging data were preprocessed using the HCP minimal processing pipelines (Glasser et al., 2013). Processing of rsfMRI data included independent component analyses (ICA)‐based denoising using FSL's multirun FIX (https://fsl.fmrib.ox.ac.uk/fsl/fslwiki/FIX), and alignment using MSMAll (Robinson et al., 2018). Further denoising was performed using the CONN Toolbox (Whitfield‐Gabrieli & Nieto‐Castanon, 2012), which included component‐based noise correction (CompCor) and band‐pass filtering. A band‐pass filter of [0.015−0.1] Hz was used to remove high‐frequency noise and low‐frequency activity with a period exceeding the duration of sliding windows while still preserving relevant low‐frequency signal, as done in prior dFCv studies (Kaiser et al., 2016; Leonardi & Van De Ville, 2015; Menon & Krishnamurthy, 2019). The high pass filters used by these prior studies support specific window lengths for sliding window analyses, since prior research suggests that the sliding window should be no less than 1/*f_min_* (the minimum frequency in the time series) to prevent spurious correlations (Leonardi & Van De Ville, 2015). Images were then converted to CIFTI space with 4 mm smoothing using the grayordinates‐based approach (Marcus et al., 2013). The quality of the functional data was assessed using relative and absolute motion plots. All subjects moved less than 3 mm, therefore, no subjects were excluded.

### 2.6 Hb and NAc seed generation

For each subject and time point, the right and left Hb were segmented using previously validated methods (Ely et al., 2019; Kim et al., 2016). In brief, as per Ely et al., the T1w, T2w, and T1/T2 ratio images from each subject were utilized to create Hb segmentations optimized for volume and shape, and time series correlations. The reliability of individual Hb segmentations was further enhanced by averaging the high‐resolution segmented Hb maps generated for each subject per hemisphere across each time point. To prevent signal contamination, signals from 3 mm spheres located in adjacent thalamic nuclei were regressed out from each Hb seed (Ely et al., 2019). The left and right NAc, which are larger structures in the brain, were segmented in standard space using the Harvard‐Oxford atlas (https://fsl.fmrib.ox.ac.uk/fsl/fslwiki/Atlases). Using the segmented and atlas‐defined Hb and NAc masks, the average time series was extracted from the denoised images for all subjects and time points to generate Hb and NAc seeds.

### 2.7 Resting‐state connectivity maps

**FIGURE 1:**
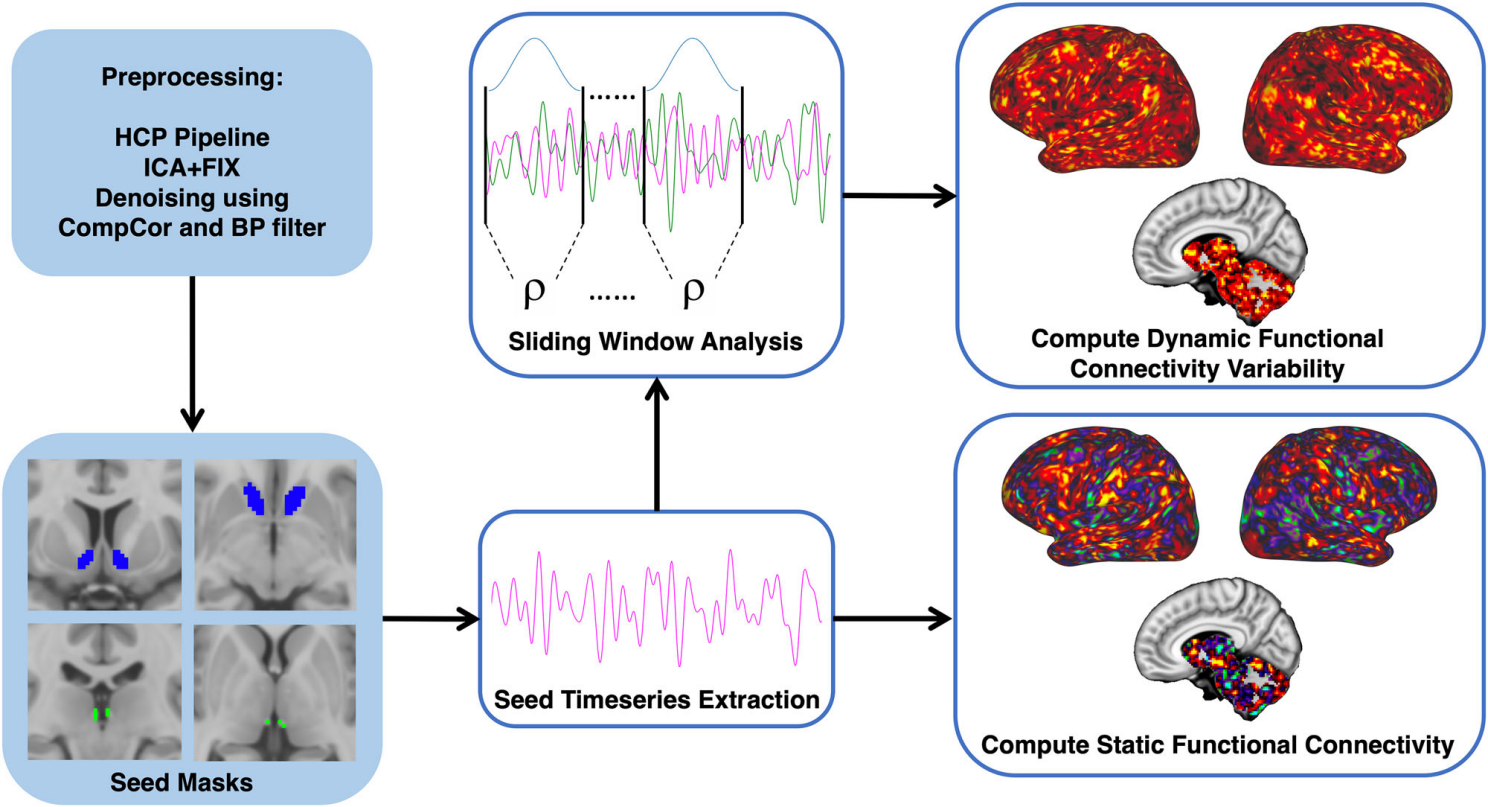
Overview of fMRI processing. Resting‐state fMRI was preprocessed using HCP protocols, which includes motion correction, correcting for EPI‐induced distortions and the conversion to CIFTI grayordinate space, and was followed by further denoising in the CONN Toolbox. Habenula masks were acquired for each subject following a validated segmentation protocol using T1w and T2w images, while the nucleus accumbens masks were obtained from the Harvard‐Oxford atlas in standard space. The average fMRI time series was extracted from each seed mask and used to generate whole‐brain functional connectivity (FC) maps. Static FC was computed directly from the extracted time series, whereas dynamic FC variability was computed after running a sliding window analysis.

The average Hb and NAc time series were used to generate seed‐based sFC and dFCv maps in CIFTI space. Seed‐based sFC maps were generated using HCP workbench commands (Van Essen et al., 2013) (https://www.humanconnectome.org/software/workbench‐command) to calculate the correlation between the time series from the seeds and every other vertex and voxel in the brain, followed by fisher‐z transformation. Seed‐based dFCv maps were calculated using the sliding‐windows analysis approach (Allen et al., 2014; Hutchison et al., 2013; Leonardi & Van De Ville, 2015), implemented using in‐house code developed in Python (https://github.com/btaraku/SeedBased_dynamic_FC). Window length is an important parameter, since sliding windows should be short enough to identify rsfMRI fluctuations, while also being long enough to avoid detecting spurious correlations. We employed similar temporal filtering and window lengths to another study using comparable HCP fMRI data (Menon & Krishnamurthy, 2019). The higher temporal resolution of HCP fMRI sequences enables us to sample a larger number of TRs within a shorter window of time compared to prior dFCv studies (Chen et al., 2022; Li et al., 2018; Zhou et al., 2021), which is advantageous since more data points decrease the influence of noise on the correlation estimates (Leonardi & Van De Ville, 2015). Here, we used a window length of 67 s (84 TRs) since this is no less than 1/*f_min_* (Leonardi & Van De Ville, 2015). Additionally, we used a Hamming window, since tapered window functions may limit detecting outlier points near window boundaries (Chen et al., 2022; Leonardi & Van De Ville, 2015; Preti et al., 2017), and used a step size of 1 TR (0.8 s). Correlations were computed across all vertices and voxels within each window and were followed by fisher‐z transformation. Once correlations were generated for all windows, the sample standard deviation was calculated across windows to calculate dFCv, which were then converted back to CIFTI grayordinate space. dFCv results were validated by trying different window lengths (100 s [125 TRs] and 50 s [63 TRs]). Figure 1 provides a flow chart of the imaging processing and fMRI analysis steps used to generate seed‐based sFC and dFCv maps in CIFTI space.

### 2.8 Statistical analyses

To examine ketamine's effect on our clinical measures of interest, paired *t*‐tests were performed to determine whether HDRS or SHAPS scores significantly changed in participants following SKI.

Whole brain group‐level analyses were conducted in PALM (Winkler et al., 2014) (https://fsl.fmrib.ox.ac.uk/fsl/fslwiki/PALM), and focused on intrasubject comparisons across all TRD patients to assess the impact of ketamine treatment. Here, we tested (1) whether longitudinal effects of FC occur following treatment and (2) if associations between FC changes and improvements in mood and anhedonia occur following treatment. All analyses were run using 5000 randomly generated permutations, using threshold‐free cluster enhancement (TFCE) and family‐wise error rate correction (FWER). Age and sex were included as covariates of no interest in all analyses. To test for longitudinal changes following SKI, paired sample *t*‐tests were performed on sFC and dFCv maps pre‐to‐post treatment at every vertex/voxel using PALM.

To test whether sFC or dFCv changes were significantly associated with improvements in depressive symptoms and anhedonia, correlations between changes in sFC and dFCv and percent change in each clinical score (HDRS, SHAPS) were calculated at every vertex/voxel using PALM. To prevent spurious correlations, outliers with extreme values (+/− 3 standard deviations from the mean) were removed prior to running PALM. As a result, one subject was removed from the HDRS correlation analysis and one subject was removed from the SHAPS correlation analysis. One additional subject was removed from both analyses due to missing clinical data. For visualization of effects, the average change was extracted from significantly correlated brain regions (TFCE/FWER corrected) and used to map associations with changes in mood on a scatter plot. Post‐hoc correlations were calculated in MATLAB using partial correlations controlling for age and sex.

As a follow‐up analysis, regions which showed significant effects in patients were assessed by comparing the average sFC or dFCv within these regions between HC and TRD at baseline, using independent two‐sample *t*‐tests. Additionally, these regions were used to assess changes in the subsample of controls scanned twice using paired *t*‐tests, to ensure that longitudinal changes observed during TRD are due to ketamine and not due to some other change in the scanning environment. Follow‐up analyses with controls were performed in MATLAB and controlled for age and sex.

## 3 RESULTS

### 3.1 Demographic and clinical results

Participants with TRD showed significant improvements in both HDRS (*t* = −13.496, *p* = 4.3e‐19), and SHAPS (*t* = 7.64, *p* = 3.27e‐10) scores following SKI treatment. Of the 58 participants who completed SKI, 29 reached remission status. Table 2 shows an overview of participant demographics and symptom changes over time following SKI.

**TABLE 2:** Demographics and clinical values by group and time point.

	HC	MDD	MDD post‐SKI	*t*/χ2	*p*‐value
Age in years: mean (SD)	32.61 (12)	40.7 (11.3)	–	*t* = 3.70	.00034
Sex: %female	56.36	48.28	–	χ2 = 0.035	.851
ISCED education variable: mean (SD)	5.96 (1.06)	5.79 (1.2)	–	*t* = −0.776	.44
Duration of lifetime illness in years: mean (SD)	–	24.78 (16.3)	–	–	–
Current episode in years: mean (SD)	–	3.69 (5.10)	–	–	–
Race (%Asian)	18.18	10.34	–	χ2 = 1.427	.2323
Race (%Black)	20	0	–	χ2 = 12.851	.0003
Race (% Hawaiian or Pacific Islander)	1.81	0	–	χ2 = 1.064	.3023
Race (% more than one race)	7.27	1.72	–	χ2 = 2.055	.1517
Race (% other/unknown/not reported)	12.73	6.9	–	χ2 = 1.093	.2958
Race (% White)	40	81.03	–	χ2 = 21.433	3.6E‐06
HDRS score: mean (SD)	–	19 (4.76)	8.4 (4.6)	*t* = −13.49	4.30E‐19
SHAPS score: mean (SD)	–	32.35 (6.79)	39.95 (7.02)	*t* = 7.64	3.27E‐10

Abbreviations: HC, healthy controls; HDRS, Hamilton Depression Rating Scale; MDD, major depressive disorder; SD, standard deviation; SHAPS, Snaith–Hamilton Pleasure Scale.

### 3.2 Effect of treatment on Hb and NAc FC in MDD

**FIGURE 2:**
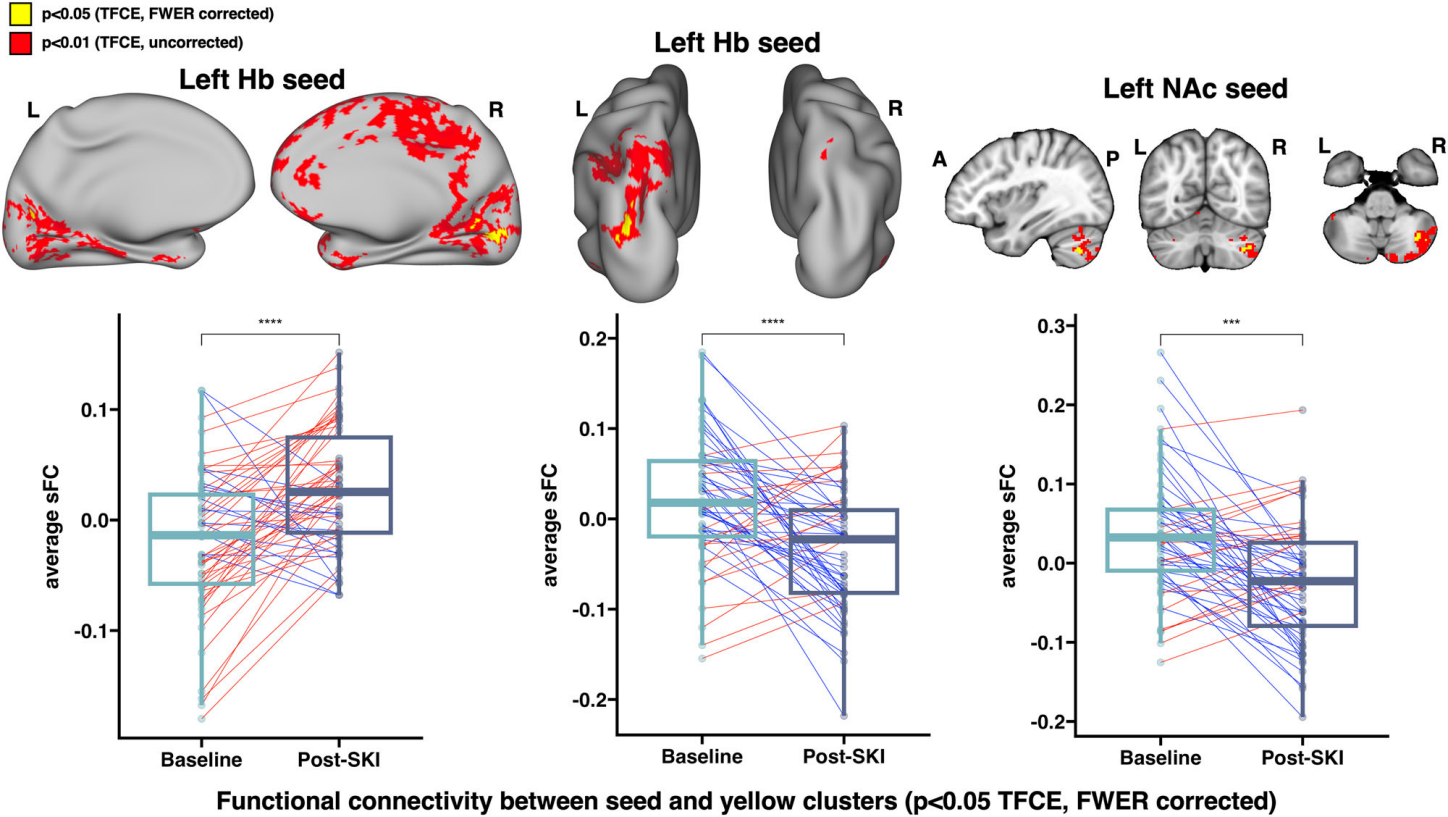
Longitudinal effects of ketamine treatment on functional connectivity. Paired *t*‐tests comparing FC change before and after ketamine treatment revealed (a) significant increases in static FC between the left habenula (Hb) and bilateral visual cortex, (b) significant decreases in static FC between the HbL and left inferior parietal cortex, and (c) significant decreases in static FC between the left nucleus accumbens (NAc) and right cerebellum (all *p* < .05, FWER and TFCE corrected). Boxplots show average static FC before and after ketamine treatment averaged within significant regions (yellow clusters) across all treatment‐resistant depression (TRD) participants. Brain images include results in surface and voxel space, and display corrected *p*‐values overlaid on top of uncorrected *p*‐values, in order to show trends. Abbreviations: FWER, family‐wise error rate; Hb, habenula; NAc, nucleus accumbens; sFC, static functional connectivity; SKI, serial ketamine infusions; TFCE, threshold‐free cluster enhancement.

Significant increases in sFC were found between the left Hb and bilateral visual cortex, as well as significant decreases in sFC between the left Hb and left inferior parietal cortex. Significant decreases in sFC were found between the left NAc and right crus I and II of the cerebellum (all *p* < .05, TFCE/FWER corrected) (Figure 2). No significant changes in dFCv were observed over time.

### 3.3 Associations between changes in FC and changes in clinical symptoms

**FIGURE 3:**
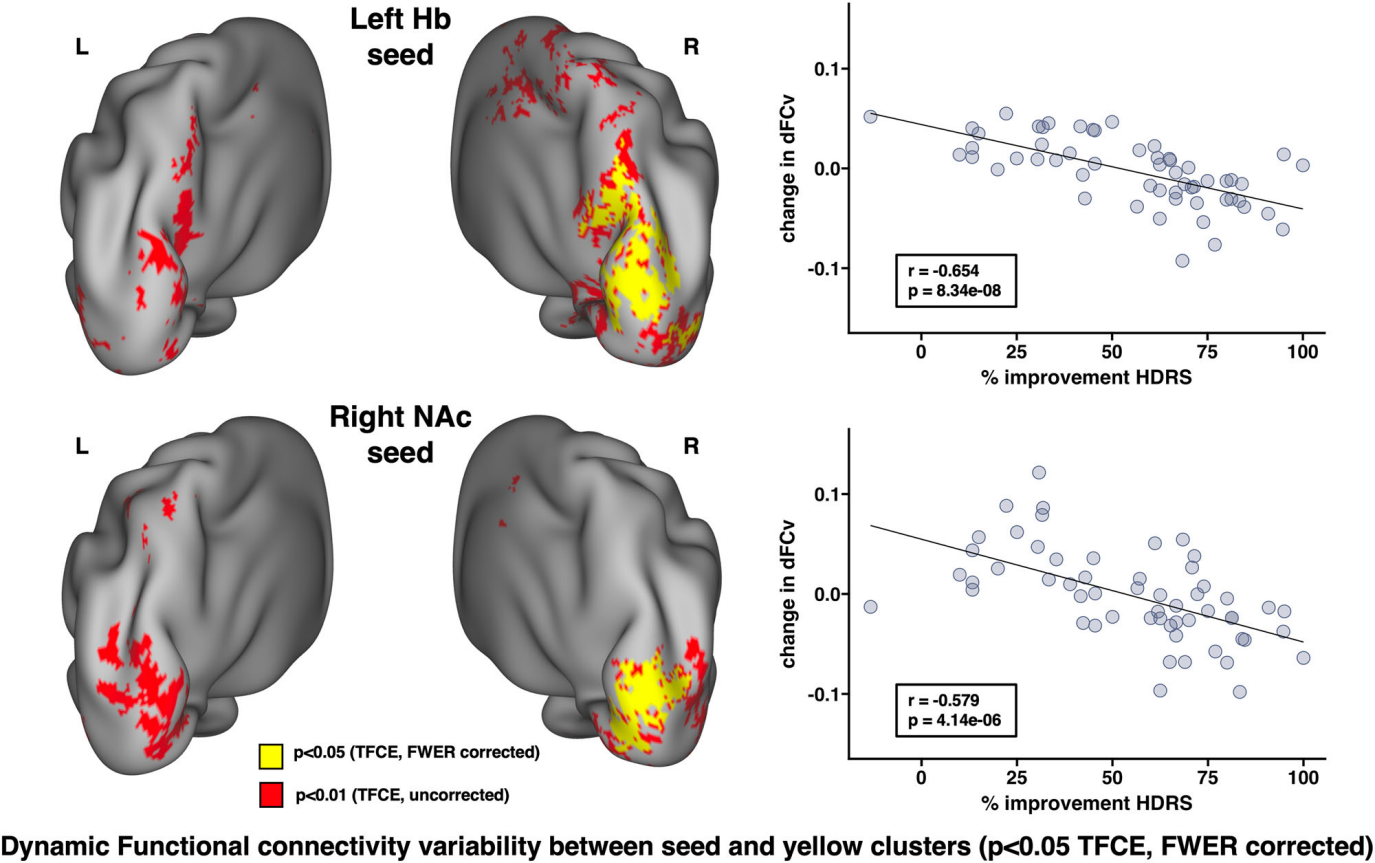
Associations between changes in dynamic functional connectivity variability (dFCv) and improvements in depressive symptoms following ketamine treatment. Whole brain correlations were performed to investigate how FC changes were associated with improvements in HDRS scores. Results revealed that decreases in dFCv between (a) the left habenula (Hb) and right precuneus and visual cortex, as well as decreases in dFCv between the (b) right nucleus accumbens (NAc) and right visual cortex were significantly associated with improvements in HDRS (all *p* < .05, FWER and TFCE corrected). Overlapping effects between the Hb and NAc were observed within a region of the right visual cortex. Average dFCv change was calculated within significant regions (yellow clusters) to create scatter plots between dFCv and HDRS change in order to visualize the overall effects within these regions. *R* and *p* values are shown on each scatter plot, and brain images show corrected *p*‐values overlaid on top of uncorrected *p*‐values to display trending effects. Abbreviations: dFCv, dynamic FC variability; FC, functional connectivity; FWER, family‐wise error rate; Hb, habenula; NAc, nucleus accumbens; TFCE, threshold‐free cluster enhancement.

**FIGURE 4:**
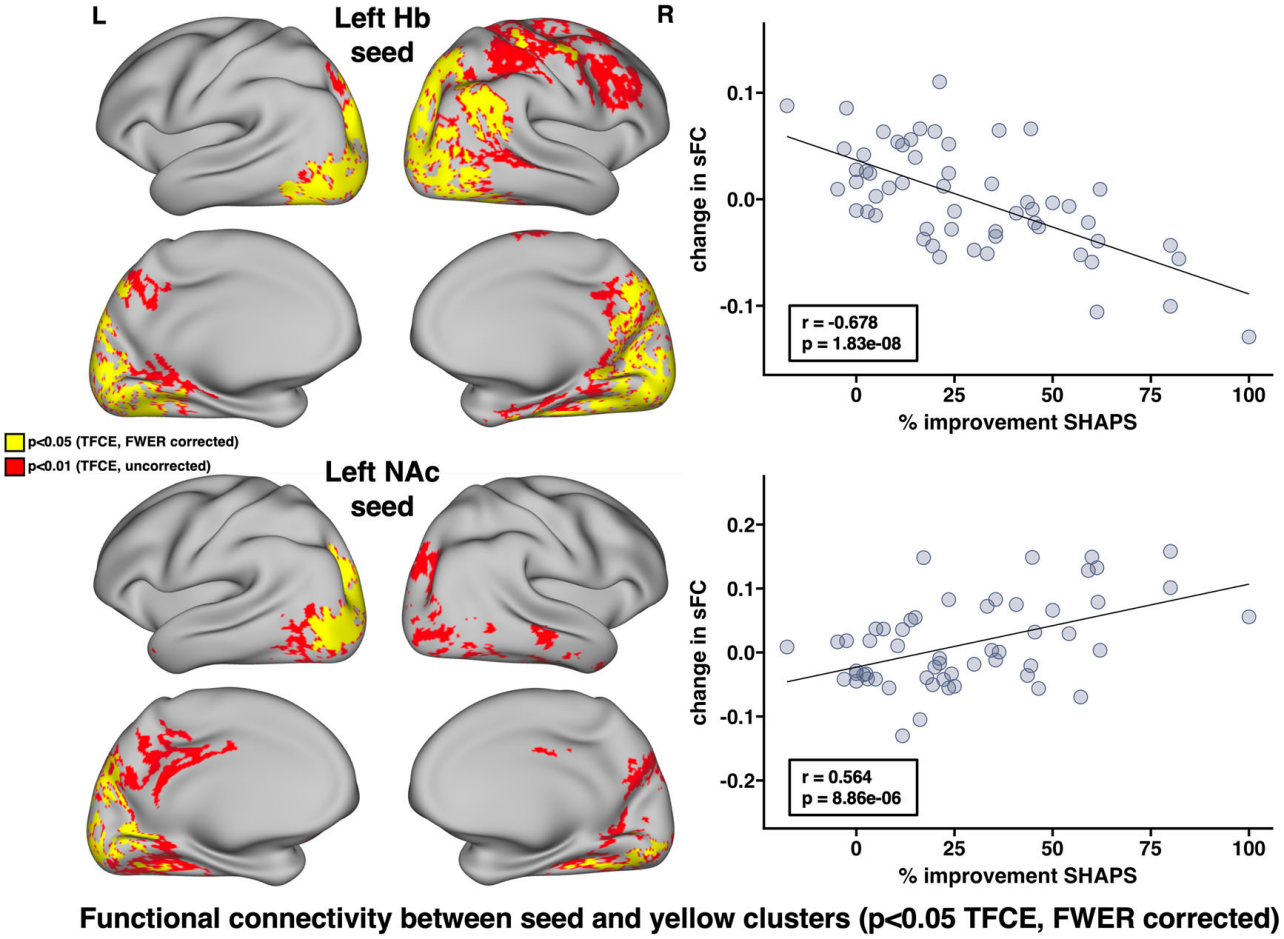
Associations between changes in static functional connectivity (sFC) and improvements in anhedonia following ketamine treatment. Whole brain correlations were performed to investigate how FC changes were associated with improvements in anhedonic symptoms using the SHAPS. Results revealed that decreases in sFC between (a) the left habenula (Hb) and bilateral visual and parietal cortex as well as the right precuneus, right temporoparietal junction, right fusiform gyrus, and right somatomotor cortex were significantly associated with improvements in SHAPS. Additionally, increases in sFC between (b) the left nucleus accumbens (NAc) and left visual and parietal cortex, as well as the right fusiform gyrus were significantly associated with improvements in SHAPS (all *p* < .05, FWER and TFCE corrected). Overlapping effects between the Hb and NAc were observed within regions of the left visual and parietal cortex, as well as the right fusiform gyrus. Average sFC change was calculated within significant regions (yellow clusters) to create scatter plots between sFC and SHAPS change to visualize the pattern of effects within these regions. *R* and *p* values are shown on each scatter plot, and brain images show corrected *p*‐values overlaid on top of uncorrected *p*‐values to display trending effects. Abbreviations: FC, functional connectivity; FWER, family‐wise error rate; Hb, habenula; NAc, nucleus accumbens; sFC, static FC; TFCE, threshold‐free cluster enhancement.

Decreases in dFCv between the left Hb, right precuneus, and right visual cortex, as well as between the right NAc and right visual cortex, were both significantly correlated with improvements in HDRS following SKI (Figure 3). The results of this analysis using different window lengths can be seen in Figure S1. Decreases in sFC between the left Hb and bilateral visual and parietal cortex, right precuneus, right angular gyrus, right fusiform gyrus, and right somatomotor cortex were significantly correlated with improvements in SHAPS following SKI. Increases in sFC between the left NAc and left visual cortex, left parietal cortex, and right fusiform gyrus were significantly correlated with improvements in SHAPS following SKI (Figure 4) (all *p* < .05, TFCE/FWER corrected).

### 3.4 Follow‐up analyses with controls

We did not observe significant cross‐sectional differences between TRD and HC at baseline within any of the regions showing significant SKI effects in our TRD sample. Furthermore, we did not observe any changes in sFC or dFCv in the subsample of longitudinal controls within any of these regions.

## 4 DISCUSSION

In this study, we examined how FC of the Hb and NAc, key nodes of reward circuitry implicated in the neurobiology of depression (Gold & Kadriu, 2019; Proulx et al., 2014; Yang et al., 2018), are perturbed by SKI treatment in participants with TRD, and associate with improvements in mood and anhedonia. Using seed‐to‐whole brain analyses to examine both static and dynamic FC, we observed significant changes in sFC following SKI that included increases between left Hb and bilateral visual cortex, decreases between left Hb and left posterior parietal cortex (PPC), as well as decreases in sFC between the left NAc and right cerebellum. Reduced dFCv between the left Hb and contralateral precuneus and visual cortex and reduced dFCv between right NAc and visual cortex were found to be significantly associated with antidepressant response. In addition, increases in sFC between the left Hb and bilateral parietal and visual regions, and reductions in sFC between left NAc and left parietal and visual regions were found to be significantly associated with improvements in anhedonia specifically. Together, these findings suggest that ketamine's therapeutic effects encompass overlapping functional circuitry of the Hb and NAc, both considered central to reward processing.

### 4.1 Effect of treatment on Hb and NAc FC

Significant increases in sFC between the Hb and the visual cortex were observed over the course of SKI treatment in TRD participants. To date, the majority of published FC studies of depression have focused on examining differences in large‐scale functional networks such as the default mode network (DMN), salience (SN), and frontoparietal/central executive (FPN/CEN) and limbic networks. However, alterations in visual networks are frequently associated with depression (Lu et al., 2020; Zeng et al., 2012). Further, three studies from our own group employing different imaging modalities and including an overlapping TRD sample have reported ketamine‐related modulation of visual areas, including changes in BOLD activation during a response inhibition task (Sahib et al., 2020), increases in cerebral blood flow (Sahib et al., 2020), and changes in white matter microstructure within occipital white matter pathways (Taraku et al., 2023). Notably, one of the few published studies investigating the effects of ketamine on Hb FC found that increases in sFC between the Hb and occipital pole and lateral visual cortex following a single ketamine infusion were significantly associated with improvements in subjectively rated depressive symptoms (Rivas‐Grajales et al., 2021). In addition, another study observed reduced sFC between the left Hb and right calcarine gyrus within the visual cortex in depressed patients and controls (Zhu et al., 2023), suggesting that the changes we observed may normalize these observed differences. Another prior study found that MDD participants exhibited decreased long‐range FC density in several regions of the visual cortex compared to controls, highlighting that the visual cortex may be an important network hub for monitoring MDD treatment effects (Zou et al., 2016). Thus, our findings in conjunction with prior research suggest that ketamine's antidepressant effects may contribute to changes in visual and reward networks.

We also observed decreases in sFC between Hb and inferior parietal lobule (IPL) following ketamine treatment in TRD participants. The IPL is contained within the PPC (Whitlock, 2017), which forms a key node within the frontoparietal network (FPN). The FPN is involved in the top‐down regulation of attention and emotion and nodes within the FPN generally show hypoconnectivity in depression (Kaiser et al., 2015). Thus, perturbation of Hb‐FPN circuitry by ketamine may contribute to its antidepressant effects.

Decreases in sFC between the NAc and the right Crus I and II regions of the cerebellum were also observed pre‐to‐post ketamine in TRD participants. Although the cerebellum is classically viewed as involved in motor coordination, much evidence shows the cerebellum also contributes to cognitive and emotional function (Adamaszek et al., 2017; Wagner & Luo, 2020). fMRI studies suggest the cerebellum and basal ganglia are strongly interconnected, forming a network involved in reward and learning (Bostan & Strick, 2018). For example, activation in the cerebellar Crus I has been associated with emotion processing, and activity in both the Crus I and II have been linked with executive function in task fMRI studies (Beuriat et al., 2022), suggesting that these regions of the cerebellum may be involved in higher cognitive functions relevant to depression. Studies also suggest that Crus I and II form connections with the FPN and DMN (Depping et al., 2018), further implicating these regions’ involvement in cognitive and emotional processing. Furthermore, several studies have found abnormalities in cerebellar circuitry in depressed participants (Dai et al., 2022; Liu et al., 2010; Minichino et al., 2014; Xu et al., 2017), which includes reduced gray matter density in Crus I in MDD patients compared to controls, which was also correlated with greater depressive symptoms in patients (Xu et al., 2017). In addition, we have previously found that ketamine treatment induces functional changes in the cerebellum during a response inhibition task‐fMRI (Loureiro et al., 2021). These findings thus further implicate the cerebellum's involvement in major depression, and suggest cerebellar‐striatal circuitry as a potential target for antidepressant treatments.

### 4.2 Associations with clinical response

Since longitudinal changes may be the consequence of biological effects independent of antidepressant effects, we examined whether changes in Hb and NAc circuitry are associated with improvements in depressive symptoms and anhedonia. We found that changes in both sFC and dFCv were associated with improvements in overall mood and anhedonia. While sFC measures temporal correlations and can determine whether there is a strong or weak excitatory or inhibitory relationship between brain regions, dFCv determines the degree of fluctuations in connectivity strength between brain regions, which are also reported as relevant to psychiatric disease states (Chen et al., 2022; Gao et al., 2022; He et al., 2018; Kaiser et al., 2016; Qiao et al., 2020; Rolls et al., 2021; Zhou et al., 2021). Prior studies have reported differences in dynamic FC from the Hb (Qiao et al., 2020) and striatum (Chen et al., 2022; Zhou et al., 2022) in depressed participants when compared to HC, further highlighting dynamic alterations in reward circuitry as a potential biomarker for MDD. Although several previous studies have investigated dynamic FC alterations in MDD within reward circuitry, no previous studies to our knowledge have investigated how dynamic FC is altered in Hb or NAc networks in MDD following ketamine treatment.

Our findings show that greater decreases in dFCv between Hb, precuneus, and visual cortex, as well as between NAc and overlapping regions of the visual cortex, are associated with greater treatment response to ketamine. Although we also found that ketamine treatment significantly increases sFC between Hb and visual cortex, these findings together do not necessarily conflict. Rather, decreases in dFCv imply less temporal variability in connectivity strength, which suggests a convergence toward a stable connectivity value within these networks as depressive symptoms improve. Given the small overlap between the regions from these two findings, one possible interpretation could be that increases in Hb‐visual cortex sFC following ketamine treatment may lead to downstream effects which reduce FC variability across broader regions of the visual cortex. One study investigating ketamine's effect on dynamic FC in nondepressed HC using whole brain atlases found that ketamine produces an overall negative effect on dynamic FC within visual networks (Spies et al., 2019), which complement our results. Since we observed associations in overlapping regions of the left visual cortex for both Hb and NAc seeds, this suggests that dynamic visual cortex activity may be involved in reward circuitry disturbances, and ketamine's antidepressant effects could stabilize activity between these visual and reward networks. Further research is necessary to understand the precise biological mechanism driving the interplay between visual and reward systems that may underlie ketamine's antidepressant effects.

Furthermore, our findings implicate decreased dFCv between the Hb and precuneus with therapeutic response to ketamine. The precuneus is a key node of the DMN, which has been widely implicated in major depression (Liston et al., 2014; Marchetti et al., 2012; Sheline et al., 2009), and is often associated with self‐referential thinking and ruminative symptoms of depression (Hamilton et al., 2015; Zhou et al., 2020). Dynamic FC disruptions in the DMN have been reported in MDD, including studies that have found decreases in dFCv within core DMN components (Demirtaş et al., 2016; Kaiser et al., 2016), and increases in dFCv between DMN and other brain regions including dlPFC and insula, which also correlated with symptom severity (Kaiser et al., 2016) in depressed individuals compared with controls. However, another study reported increased dFCv within the core DMN components in depressed participants compared to controls (Wise et al., 2017). Of relevance to this study, greater dFCv between the Hb and right precuneus was found in depressed participants with suicide ideation compared to HC, although opposite trends were found for the contralateral precuneus (Qiao et al., 2020). Interestingly, the precuneus is also suggested to be involved in the dissociative effects induced by ketamine, where reductions in oscillations measured by electroencephalography in the precuneus are associated with dissociation (Tian et al., 2023; Vlisides et al., 2018). Thus, it may be the case that ketamine's antidepressant effects and dissociative effects are driven by similar neural mechanisms. In conjunction with prior findings, our results suggest that ketamine's antidepressant effects may modulate dynamic FC disruptions between the Hb and precuneus.

Decreases in sFC between Hb and posterior parietal and visual cortex, angular gyrus, precuneus, motor cortex, and fusiform gyrus, as well as increases in sFC between NAc and overlapping regions in the visual cortex, parietal cortex, and fusiform gyrus were associated with improvements in anhedonia. The NAc forms an integral part of the brain's reward circuitry and shows hypoactivity in depression, whereas the Hb is part of the antireward system and shows hyperactivity in depression. Our results suggest the antianhedonic effects of ketamine may reverse patterns of aberrant reward circuitry commonly seen in depression, by increasing NAc and subsequently decreasing Hb connectivity in both broadly distributed overlapping, as well as and distinct brain regions. The overlapping regions occurred primarily in the visual cortex, PPC, and fusiform gyrus. Relevant to our findings, a prior study found reduced FC between NAc and IPL in participants with depression and also found a significant interaction between anhedonia and diagnosis of FC between NAc and IPL (Liu et al., 2021), highlighting parietal cortex contributions to anhedonic symptoms.

In addition to visuo‐parietal regions, significant decreases in FC between Hb and the precuneus and angular gyrus, key nodes of the DMN, were correlated with improvements in anhedonia. Of relevance to this finding, a prior study reported hyperconnectivity between the Hb and precuneus which was associated with suicidality in TRD (Barreiros et al., 2022). Thus, our results suggest greater response to ketamine normalizes hyperconnectivity between Hb and DMN. Decreases in Hb and somatomotor cortex FC were also found to be significantly associated with improvements in anhedonia. Prior imaging studies have reported altered FC of somatosensory and somatomotor networks correlate with depressive symptoms, including suicidality (Fan et al., 2022; Kang et al., 2018). Preclinical studies also suggest that Hb inhibition of dopamine neurons leads to the suppression of body movements and to lowered motivation (Hikosaka, 2010).

### 4.3 Cross‐sectional analysis

We failed to observe cross‐sectional differences between HC and TRD at baseline, which hinder our interpretation of whether any observed treatment effects trend toward values seen in nondepressed individuals. Despite this, other studies have reported cross‐sectional differences in Hb and NAc FC between controls and depressed patients across regions implicated in our study. This includes reduced sFC between Hb and visual cortex in MDD patients compared to controls (Zhu et al., 2023), greater sFC between Hb and precuneus in TRD compared to controls (Barreiros et al., 2022), and a study which found increased sFC between contralateral NAc and cerebellum in adolescent MDD patients compared to controls (Chen et al., 2023). Therefore, these prior findings suggest the SKI‐induced changes observed in our TRD sample may trend toward connectivity patterns typically seen in controls relative to depressed patients.

### 4.4 Limitations

We acknowledge that there are several limitations associated with the current investigation. First, the Hb is a small structure, and the spatial resolution of imaging protocols may still be inadequate for fully capturing structural and functional changes in this region even when using advanced acquisition and preprocessing methods. On a related note, we were also not able to reliably assess change and relationships between the NAc and Hb with brain stem nuclei, noting that recent findings suggest increased FC between the Hb and the substantia nigra and VTA associate with improved depression scores following SKI (Chen et al., 2024). We also note that the current study utilized a naturalistic study design and did not include randomization to a placebo condition. However, this research study was designed as a mechanistic clinical trial focused specifically on imaging outcomes for which subjects had no expectations rather than on the efficacy of SKI. Further, we did not observe differences between patients and controls at baseline; however, we note that this study was not specifically powered to perform cross‐sectional analyses between diagnostic groups. On the other hand, our investigation of changes within controls across time suggests that the observed changes are associated with ketamine treatment. Finally, participants were allowed to continue concurrent stable antidepressant medication, which may have impacted findings although we note that participants serve as their own controls in longitudinal analyses and did not change medication throughout the trial.

## 5 CONCLUSION

Serial ketamine treatment in TRD modulates static and dynamic FC in both overlapping and distinct Hb and NAc functional networks. SKI produces distinct static FC changes from Hb and NAc seeds in TRD participants, whereas static and dynamic FC changes associated with clinical response occurred in broader overlapping regions from Hb and NAc seeds, suggesting that antidepressant response to SKI involves reward, sensory, and self‐referential networks.

## AUTHOR CONTRIBUTIONS

KN, AL, JL, RE contributed to the overall study design and data acquisition. BT and JL conceived the current research, performed the computational imaging and statistical analyses. AS and BW contributed to research design and data processing. JL and BT wrote the first draft of the manuscript, and BT made subsequent drafts of the manuscript and made the figures. All authors (BT, JL, AS, AZ, NS, AL, BW, SJ, RP, RE and KN) assisted with the interpretation of results and scientifically contributed to final manuscript editing and preparation.

## CONFLICT OF INTEREST STATEMENT

The authors have no conflict of interest to report.

### PEER REVIEW

The peer review history for this article is available at https://publons.com/publon/10.1002/brb3.3511.

## Supporting information


**Figure S1**: Associations between changes in dFCv and HDRS after modifying window length.

## Data Availability

The data that support the findings of this study are publicly available in the NIMH Data Archive in collection 2844 at https://nda.nih.gov/edit_collection.html?id=2844
